# Ophthalmology

**Published:** 1877-02

**Authors:** 


### V. Ophthalmology.

1. *A case of Acute Glaucoma, caused by one single instillation of Atropia.* Magnus. (*Klin. Monatsbl*, XIV, p. 386.)

A healthy farmer, aged 74 years, applied for the extraction of cataract. The examination showed a mature senile cataract in both eyes, normal anterior chamber, normal mobility of the iris, normal tension of the eyeball and a precise perception of light in every part of the visual field. As the patient was ready to undergo the operation the following day, Dr. M. put a drop of a solution of atropiæ sulphas in each eye, because he wished to examine carefully the cataracts when the pupil would be fully dilated. The patient, who lived in a suburb, returned home after the consultation. The next morning, however, he did not come back to town as he intended, but Dr. M. received a note from the farmer's wife, informing the doctor that about five hours after the instillation of atropia, the right eye had become very red and painful, and the patient was suffering such pain in the right side of his head that he was unable to leave the bed. Dr. M. suspecting the possibility of glaucoma, at once sent back the urgent order to bring the patient to him without delay. But the patient did not come till three weeks later; he had suffered the most agonizing pain all the while. The right eye showed the typical picture of acute glaucoma in that stage when the inflammatory attack is on the decrease. A complete dilatation and immobility of the pupil, a shallow anterior chamber, a marked vascular injection around the cornea, a great increase of the hardness of the eyeball, and a considerable limitation of the per ception of light, the patient being able to perceive a large light only when held right in front of the right eye. The left eye showed the same condition as at the first consultation.

2. *Foreign body in the eyeball for fourteen years without disturbing the sight.* Martin. (*Receuil d'Ophthalmologie*, Oct. 1876.)

About five years ago an employee at a marble work presented himself to the doctor with pain, photophobia, lachrymation and redness of the left eye. He stated that every spring and fall of a good many years past, without any appreciable cause, he had been seized by similar attacks which caused him much pain and confined him to the room during a whole month.

The focal examination detected the presence of a dark rounded body, not larger than the head of a pin, resting on the crystalline lens and riding on the inferior outer border of the pupil. When the patient was informed of the presence of a foreign body in his eye, and he had given this matter a few moments' thought, he distinctly remembered that the first inflammation of his eye followed a contusion of the same by a piece of marble, that happened fourteen years ago, and ever since then the inflammation recurred every year. No sign of lesion could be found in the cornea. For the removal of the foreign substance, a broad linear incision was made through the lower external margin of the cornea and the aqueous humor was allowed to escape very slowly for fear lest the foreign body would drop down behind the iris. With a pair of forceps that body (which proved to be a triangular piece of black marble,) was easily seized and extracted. The iris protruding a little through the wound, a portion of it was excised to prevent the formation of a hernia of the iris. The pain left the eye, the wound healed kindly, the eye has never since been troubled and still has a perfect vision. It is very remarkable that in spite of those frequent attacks of recurrent iritis there were neither any posterior synechiæ nor any exudition on the anterior surface of the lens.

3. *Blepharoraphia Medialis.* Arlt. (*Wiener Med. Wochenschr.* 40, 1876.)

Under this name Prof. A. describes an operation for shortening the palpebral aperture from the inner canthus as the tarsoraphy does from the external canthus. He pares the free border of each lid from the lachrymal punctum to the inner canthus. The wounds thus established measure two to three millimetres in width and seven millimetres in length ; they meet at the inner canthus in an acute angle like the two lines of the letter V. Particular attention must be paid to a careful paring of this junction of the two wounds, else first union will not take place there, and an annoying fistula will remain. The slight bleeding arrested, three stitches generally are sufficient to bring the pared edges in perfect coaptation. To secure a kind healing the occlusion of the eye by a bandage during two or three days is advisable. Prof. A. finds this operation especially useful in cases of eversion of the lower lid, caused by paralysis of the facial nerve. In those cases the lower lid (especially its inner or nasal portion) has sunken down a little and does not perfectly hug the eyeball any more. The sequelæ of this displacement of the lid, are epiphora and irritation or inflammation of the conjunctiva and cornea.* In two such cases, Prof. A has performed the above operation with this result, that the lower lid was raised and its border was brought nicely in contact with the eyeball; and that with the readjustment of the lid the epiphora and other sequelæ were efficiently stopped.

